# Gatekeeper competencies for suicide prevention among Korean social work students: a path model of the relationship between education and gatekeeper intention

**DOI:** 10.3389/fpubh.2025.1630381

**Published:** 2025-08-14

**Authors:** Jungyai Ko

**Affiliations:** Department of Social Welfare, Hallym University, Chuncheon, Republic of Korea

**Keywords:** suicide prevention, gatekeeper training, social work education, competencies, behavioral intention

## Abstract

**Purpose:**

With the high suicide rate and low utilization of mental health services in Korea, gatekeeping is a crucial public health strategy for suicide prevention. Social workers have the potential to serve as professional gatekeepers in this context. This study examined the relationships between social work students’ educational experiences, suicide prevention competencies, including knowledge, attitudes, and perceived preparedness, and their intentions to perform gatekeeper roles.

**Methods:**

The online survey collected data from 18 randomly selected social work schools across Korea. The final data included 496 social work students (193 in the 3rd and 303 in the 4th year). Path analyses were conducted on a hypothesized path model in which students with more educational experience had higher competencies and stronger intentions for gatekeeper roles. It was also hypothesized that these competencies mediated the relationship between educational experiences and gatekeeper intentions.

**Results:**

The results demonstrated the validity of the suggested path model (CFI = 0.995, TLI = 0.977, RMSEA = 0.027, SRMR = 0.022). Classroom learning was associated with increased knowledge about suicide (*p* < 0.05), and field experiences were related to more positive attitudes toward suicide prevention (*p* < 0.01). Students’ intention to perform gatekeeper roles was predicted by knowledge (*p* < 0.001), attitudes (*p* < 0.05), and perceived preparedness (p < 0.001), and indirectly by educational experiences.

**Conclusion:**

These findings suggest that university-level education can play a critical role in preparing social work students as professional gatekeepers by providing various educational experiences and enhancing the key competencies needed for suicide prevention. Future studies should consider adopting a longitudinal design to understand the association between education and actual practice behavior.

## Introduction

Causing more than 720,000 deaths annually, suicide is a global public health problem (1). The multidimensional nature of the causes of suicide makes it essential for suicide prevention strategies to adopt comprehensive approaches across sectors (2). Among the various strategies, increasing the number of people with suicide assessment and risk management skills is considered one of the most effective methods for suicide prevention (3, 4). Accordingly, gatekeeper training has been widely adopted in many community suicide-prevention strategies (5, 6). The term “gatekeeper” refers to individuals in a community who regularly have face-to-face contact with large numbers of community members and can identify persons at risk of suicide and warning signs and refer them to appropriate services (7). Social work professionals have the potential to engage in the gatekeeper role; however, there is a lack of empirical evidence on their educational experiences and readiness as gatekeepers for suicide prevention. Thus, this study explores the relationships between social work students’ educational experiences regarding suicide, their competencies, and their intent to engage in a gatekeeper role for suicide prevention in Korea.

### Suicide and suicide prevention in Korea

The suicide rate in South Korea was 25.2 per 100,000 people, which can be compared with the global average suicide rate of 9.2 (8, 9). The suicide rate in South Korea has been the highest of all countries in the Organization for Economic Co-operation and Development (10) for the last few decades, raising social awareness for suicide prevention. Recently, the focus has been on the country’s lack of mental health help-seeking (11, 12). In 2021, the mental health service utilization rate in South Korea, defined as “treatment or counseling received from any mental health professionals, including psychiatrists,” was 7.2%, and this was significantly lower than in the US (43.1%) and Canada (46.5%). Even in cases of individuals diagnosed with a mental disorder, only 12.1% had ever utilized mental health services in their lifetime. Barriers to mental health services utilization in Korea include stigma regarding mental health problems and low accessibility to mental health services (11). Accordingly, gatekeeping approaches are gaining more attention at the community level to increase mental health service utilization (12). A meta-analysis of the effectiveness of suicide gatekeeper programs in South Korea (13) showed that gatekeeper training improved knowledge and attitudes related to suicide prevention, although its long-term effects and those on gatekeeper behavior were not sufficiently explored (13).

### Social work and social work education in suicide prevention

Suicide is an important social problem for social workers. Social workers work with diverse populations who experience a range of psychosocial challenges that also elevate the risk of suicide (14). Beyond the high likelihood of working with clients at risk, social work professionals embrace the core social work values of prioritizing the inherent dignity of human life (15), which underscores suicide prevention as a fundamental goal of social work intervention. In addition, the holistic perspective of social work practice provides a background for social workers to address the diverse factors associated with suicide risk (16).

However, definitions or guidelines are lacking regarding the specific roles or responsibilities of social workers in suicide prevention efforts. This ambiguity is particularly relevant in countries like Korea, where social workers are primarily involved in community-based rather than mental health settings (17, 18). Furthermore, pertinent to this discussion, concerns have also been raised about the lack of attention given to suicide prevention in social work education (14, 19).

In the gatekeeper model, social workers can be conceptualized as professional gatekeepers. Professional gatekeepers are trained to identify individuals at risk of suicide, assess their level of risk, provide brief interventions, and manage immediate risk by developing safety plans and referring individuals to behavioral professionals (4). While they may not provide the same level of mental health interventions as behavioral health professionals, the role of professional gatekeepers can extend beyond those of most community gatekeepers (4, 20), such as teachers or other community members (21). As social workers work in various community settings, they are likely to encounter clients at risk of suicide (16). Therefore, their education includes content related to psychosocial assessment and intervention (22). Accordingly, the definition of professional gatekeepers can help establish minimum standards for the role of suicide prevention among social workers working in community settings.

Social work education at the university level can play a critical role in training professional gatekeepers for suicide prevention. Universities can provide advantages for suicide education due to fewer time constraints and access to educational resources. Improving the competencies of professional gatekeepers involves the transformation of attitudes, perceptions, and self-efficacy, which requires time and various educational approaches (23). However, most gatekeeper programs are typically delivered through workshops with time limitations (57). Unlike general gatekeeper training programs that target a broad audience of various professional groups and the general population, universities can provide specific training content for students of particular majors (24). For instance, social work academic programs can cover how ethics, knowledge, and skills in social work practice can be translated directly to suicide prevention.

However, empirical support is lacking on how academic social work education is preparing social work students as professional gatekeepers. In fact, the gap between educational needs and opportunities has been continuously reported in terms of suicide prevention (14, 19, 25). Therefore, before discussing the relationships between social work education and professional gatekeepers, how existing educational opportunities are related to their competencies as professional gatekeepers must first be understood (16, 26).

### Gatekeeper competencies

Gatekeeper competencies refer to the core components of the capabilities required to effectively identify, assess, and refer individuals at risk of suicide (4, 23). These competencies include (1) knowledge of suicide trends, warning signs, risk and protective factors, and referral resources; (2) positive attitudes toward suicide prevention, which refers to the extent to which someone believes that suicide is preventable and that suicide prevention is essential; and (3) self-efficacy or perceived preparedness to engage in suicide prevention behaviors, that is, an individual’s belief in their ability to identify, care for, and facilitate treatment for a person at risk of suicide (23, 27).

Numerous studies have demonstrated the effectiveness of gatekeeper education in increasing knowledge about suicide (20, 24, 28–31). Furthermore, knowledge interacts with other components of suicide prevention competencies, such as attitudes and confidence, to influence intervention behavior positively (30, 32). Research also has shown that training yielded more positive attitudes toward suicide prevention (27). Attitudes also mediate the relationship between training effects and behavioral outcomes (5, 27). The concept of perceived control, or self-efficacy as defined by Bandura (33), refers to an individual’s judgment of how well they can execute actions to handle potential situations. Self-efficacy can be compared to diverse terms in the gatekeeper literature, including perceived preparedness, confidence, self-efficacy, and sense of control (32). Even if practitioners possess the necessary skills for suicide risk assessment, low self-efficacy can prevent them from adequately performing assessments (34).

### Theoretical framework for a path model

It is difficult to measure the extent to which trainees are skilled in performing gatekeeper behaviors, as assessing skill-based practice behavior is challenging without observing client data (4). Furthermore, the relatively low incidence of suicide means newly trained gatekeepers have limited opportunities to engage in gatekeeper behavior in the practice setting, making it harder to measure actual practice behavior (5). Accordingly, in the gatekeeper literature, researchers are urged to consider measuring close constructs such as intention to practice as a proximal outcome.

The theory of planned behavior (TPB) proposes that attitudes and personality traits indirectly influence certain behaviors through closely related factors such as intention (58). TPB identifies three critical predictors of intention: attitudes, subjective norms, and perceived behavioral control (58). Attitudes reflect one’s evaluation or appraisal of behavior as favorable or unfavorable, subjective norms relate to the perceived social pressure to perform the behavior, and perceived behavioral control refers to the perceived ease or difficulty of carrying out the behavior.

In the literature on gatekeeper training, TPB is frequently used to examine the relationship between predictors of intention, behavioral intention, and practice behavior (5, 35, 36). For example, in a study of clinicians who underwent 2 days of gatekeeper training, (27) found that attitudes and self-efficacy related to suicide prevention predicted both behavioral intention and practice behaviors and recommended that training curricula address behavioral intention to increase practice behaviors. In addition, based on a systematic review of the long-term efficacy of suicide prevention gatekeeper training, Holmes and colleagues (5) emphasized the importance of addressing behavioral intention among trainees, pointing out that simply educating individuals about the importance of gatekeeper behavior is insufficient in ensuring subsequent practice behavior.

### Study aims and hypotheses

**Figure 1:**
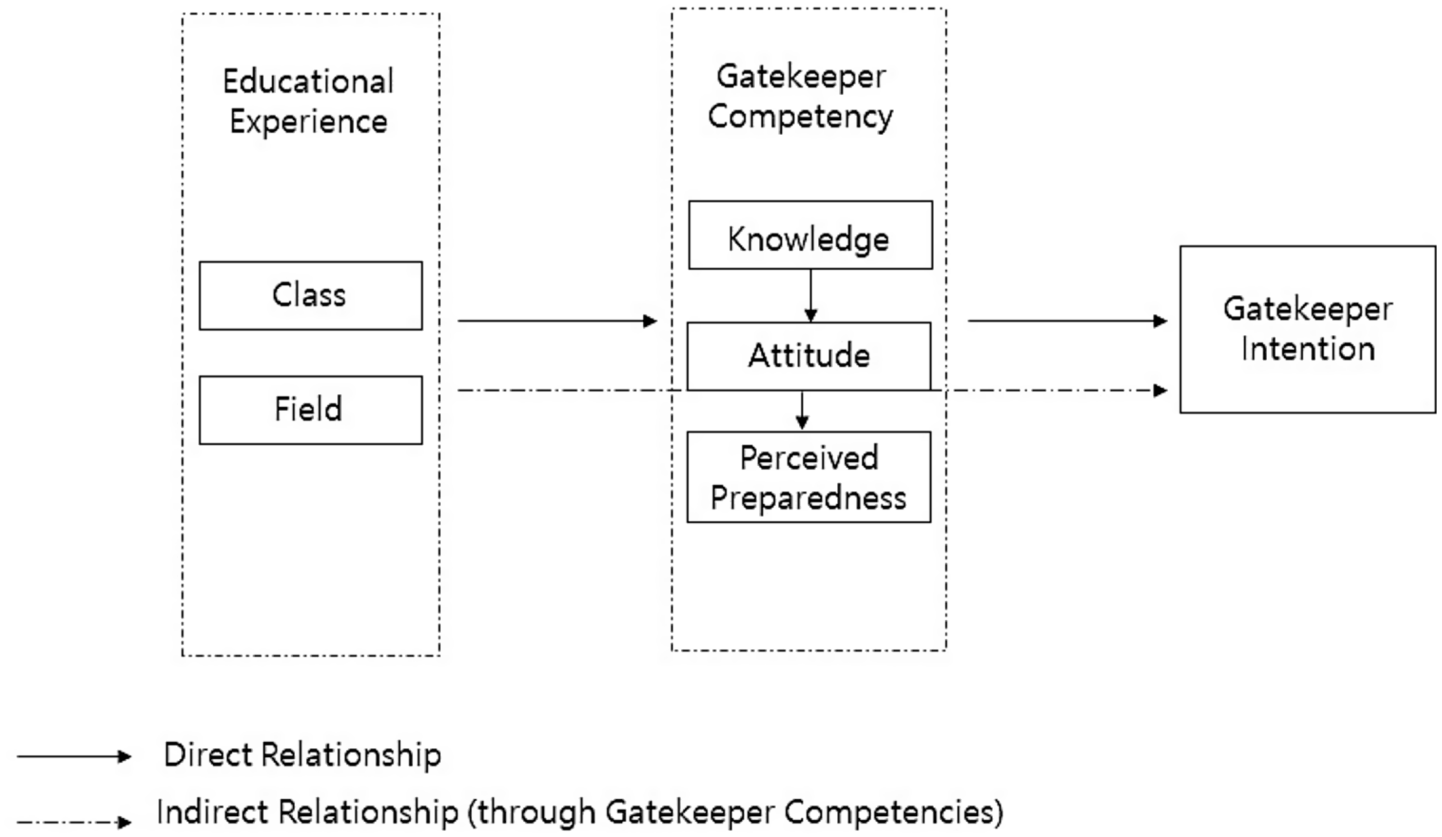
The hypothesized path model of the relationships between key variables.

Although prior studies provided support for the relationship between gatekeeper education and competencies or intentions, not many explored them within a comprehensive path model, especially among social work students. Thus, this study aims to evaluate a path model of the intention to perform gatekeeper roles among social work students and test the following hypotheses: (1) The path model (as shown in Figure 1) in which suicide-related competencies, including knowledge, attitudes, and perceived preparedness mediate the relationships between suicide-related educational experiences and the intention to perform a gatekeeper role fits among social work students. (2) Gatekeeper competencies are predicted directly and indirectly by social work students’ educational experiences. (3) Students’ intention to perform a gatekeeper role is predicted directly by suicide competencies and indirectly by educational experiences through the mediating effects of suicide competencies. The study aims to address these hypotheses and contribute to future efforts in social work education for suicide prevention.

## Materials and methods

### Data source

This study analyzed data from a nationwide online survey in Korea conducted between September and October 2020. The survey aimed to identify the educational experiences of social work undergraduate students regarding suicide and suicide prevention.^1^ The survey included questions on the students’ attitudes, knowledge, personal and educational experiences related to suicide, and their preparedness and intention to perform a gatekeeper role. The link to the survey was distributed via KaKao Talk, the most commonly used (93.5%) messaging application, which is available in Korea on PC and mobile (37). The survey was available to students for 4 weeks.

The sample was selected using cluster sampling, where 18 social work schools were randomly selected from a list of 98 social work schools provided by the Korean Council of Social Work Education (KCSWE) (38). The average number of students, 30–50 per year in each school, and the response rate for online surveys were also considered. Of the selected schools, 17 agreed to participate and distributed the online survey link to all third and fourth-year students. Before the survey questions were presented, students were given a detailed description of the survey, and only those who chose to consent by clicking “*agree to participate*” proceeded to answer the questions. The survey was sent to 1,582 students, of which 511 responded, a response rate of 32%.^2^ After excluding non-qualified respondents (e.g., those who reported being in the second year of the university or those who did not complete the field practicum), 496 responses were considered valid.

### Sample

The sample included third and fourth-year social work students who had completed their social work practicum.^3^ Table 1 presents the sample characteristics. The sample comprised 109 males (22%) and 387 females (78%). The higher proportion of female students in the current sample is aligned with prior research on social work students in Korea, and the proportion of male students falls within the range reported in previous studies [18–34%, (39, 40)]. Among the sample, 193 students reported being in the third year of the university (38.9%). The average age of participants is 22.26 years (*SD* = 1.548), with a range of 20 to 30 years. When asked about family income, 218 students reported being in the middle class (44%), and 151 reported being in the lower-middle class (30.4%). More than half the students reported having very good or good mental health (13.1% for *very good* [*n* = 65]; 38.1% for *good* [*n* = 189]). See Table 1 for additional sociodemographic characteristics of the sample. Among the sample, 326 (65.7%) students reported having learned about suicide at least once in class, and 120 learned about suicide at least once through field experiences (24.2%). Only 38 students (7.7%) reported having worked with suicidal clients at the field practicum.

**Table 1:** Sample characteristics and the results from *T*-tests and ANOVA.

Classifications				Knowledge		Attitude		Perceived Preparedness		Intention	
		*N*	*%*	*M*	*SD*	*M*	*SD*	*M*	*SD*	*M*	*SD*
Gender	Male	109	22	59.89	14.28	34.76	6.76	4.57	0.91	4.23	0.54
	Female	387	78	63.42	14.09	34.29	6.06	4.17	1.02	4.14	0.54
	*t*			−2.299^*^		0.692		3.719^***^		1.449	
School year	Third	193	38.9	61.14	14.17	35.05	6.31	4.21	1.00	4.12	051
	Fourth	303	61.1	63.60	14.14	33.98	6.13	4.29	1.01	4.18	0.55
	*t*			−1.889		1.877		−0.850		−1.329	
Family income	Upper^a^	4	0.8	67.86	9.22	23.25	4.99	4.88	0.34	4.75	0.29
	Upper-Middle^b^	85	17.1	64.03	14.18	32.71	6.41	4.37	1.02	4.29	0.53
	Middle^c^	218	44.0	62.25	13.60	34.69	5.85	4.26	1.05	4.16	0.50
	Lower-Middle^d^	151	30.4	62.49	14.97	34.99	6.37	4.21	0.97	4.10	0.55
	Lower^e^	38	7.7	61.84	15.15	35.29	5.78	4.20	0.91	4.07	0.63
	*F*			0.413		5.660^***^		0.766		3.197^*^	
	*Tukey*			n/a		a > b,c,d,e		n/a		n/a	
Mental health status	Very good^f^	65	13.1	60.55	13.66	31.28	6.92	4.45	1.10	4.30	0.55
	Good^g^	189	38.1	64.02	13.27	33.85	5.56	4.33	0.99	4.18	0.56
	Average^h^	184	37.1	62.38	15.41	35.17	5.96	4.10	0.99	4.11	0.52
	Bad^i^	46	9.3	62.42	13.08	37.07	6.36	4.26	1.00	4.04	0.47
	Very bad^j^	12	2.4	57.14	14.92	37.83	7.63	4.59	0.75	4.29	0.49
	*F*			1.271		8.731^***^		2.329		2.276	
	*Turkey*			n/a		f > h, I, j		n/a		n/a	

*p < 0.5; **p < 0.01; ***p < 0.001. The superscript in Table 3 does not need an additional footnote. It is a device used to present the results of the post hoc Tukey analysis within the table and does not carry any additional meaning. Uppera means upper itself in post-hoc Turkey analysis.

### Measures

The Korean translation of the attitudes, preparedness, and intention measures was conducted using the process suggested by Beaton et al. (41). First, the initial translation was performed by three professors from social work, English, and psychiatric nursing, all of whom hold doctoral degrees in the US and are fluent in English and Korean. After sharing their independently translated Korean versions, the differences were cross-checked to ensure cross-cultural adaptation and the face validity of the measures through multiple online meetings and email communication until the researchers reached a consensus. Next, back translation was undertaken by a bilingual licensed social worker who also holds a doctoral degree and runs a counseling center in the US. Last, a pilot test was conducted with six undergraduate social work students. Participants were recruited via the online bulletin board of the principal investigator’s university. The research team, who are the principal investigator and doctoral-level research assistants, hosted two separate Zoom meetings in August 2020. One group used a PC, and the other used a mobile device to complete the survey. Participants confirmed that there were no difficulties in understanding and responding to the survey questions. The response time was also within expectations, ranging from 12 to 13 min.

#### Exogenous variables: suicide education

To measure the students’ educational experiences related with suicide, two items were used. The first item measured their experiences in the classroom during the coursework. The second measured their experiences learning about suicide through field activities, which included a field practicum, voluntary work, and community service or organizational activities. Both items measured whether students had learned about suicide in six areas, including suicide risk assessment, suicide warning signs, referral, short-term intervention, suicide prevention policies, and suicide theories. These areas were based on professional gatekeeper roles defined by Osteen et al. (4) and suicide-related content in the KCSWE course guidelines. For the path analysis, the total number of areas in which students reported learning was calculated.

#### Mediating variables: gatekeeper competencies

##### Knowledge

For knowledge of suicide, a 14-item scale called the Warning Signs and Intervention Behaviors was used. This multiple-choice questionnaire assessed respondents’ level of knowledge of suicide and suicide prevention by calculating the percentage of correctly answered items. The scale was initially developed by Wyman et al. (42) to evaluate the effectiveness of gatekeeper training for secondary school staff. It was later used to measure suicide knowledge among MSW students (20). Kim and Kim (13) developed and validated the Korean version of the scale in a study on the suicide prevention competencies of visiting nurses. The results showed that nurses who had received suicide prevention training scored significantly higher on the scale than the control group. To examine the validity of this measure compared with the existing one, it was correlated with the total score of the three-item knowledge measure developed and used to test the effectiveness of gatekeeper training by the Korea Suicide Prevention Center (43). The total scores of the two measures were significantly correlated (r = 0.315, *p* < 0.001), providing preliminary evidence of convergent validity (44, 45).

##### Attitudes

The Attitudes Toward Suicide Prevention [ASP; (46)] scale was used to measure attitudes regarding suicide. The scale consisted of 14 statements, with 12 negatively worded and two positively worded items regarding suicide prevention. Respondents rated their level of agreement with each statement on a five-point Likert scale (1 = strongly disagree, 2 = disagree, 3 = uncertain, 4 = agree, 5 = strongly agree). To indicate the degree to which respondents held negative beliefs toward suicide prevention, the total scores were summed after reverse-coding the two positively worded items. The ASP has demonstrated good internal consistency among healthcare professionals [Cronbach’s *α* = 0.77; (46)] and MSW students [Cronbach’s α = 0.75; (20)]. Cronbach’s α for the current sample was 0.72, which falls within the acceptable range (47). Confirmatory factor analysis (CFA) was conducted. The model fit indices suggest an acceptable fit for the Korean version ASP (CFI = 0.918, TLI = 0.899, RMSEA = 0.041, SRMR = 0.040), providing preliminary evidence of construct validity (48, 49).

##### Preparedness

The study used the Perceived Preparedness for Gatekeeper Role [PPGR: (42)] to measure the perceived preparedness of respondents in performing a gatekeeper role. The scale consists of eight items, each rated on a seven-point Likert scale ranging from 1 (not prepared) to 7 (quite well prepared). The mean total score of the eight items indicates how well respondents perceive themselves as prepared for the gatekeeper role. Good internal consistencies were reported for school staff (Cronbach’s *α* = 0.94) and MSW students (Cronbach’s α = 0.91). In the current sample, Cronbach’s α was 0.87, indicating good internal consistency (47). The construct validity of the Korean version of the PPGR was tested via a CFA. The results demonstrated an acceptable model fit: CFI = 0.947, TLI = 0.917, RMSEA = 0.102, SRMR = 0.046 (48, 49).

#### Endogenous variables: suicide intention

To measure the intention to perform gatekeeper behaviors, the study modified the Use of Gatekeeper Behavior with Suicidal Ideation scale (42). The original scale consisted of six items, each rated on a five-point Likert scale ranging from 1 (*never*) to 5 (*always*), asking how often the respondent has conducted each gatekeeper behavior. Good internal consistency was found for both school staff (Cronbach’s *α* = 0.94) and MSW students (Cronbach’s *α* = 0.93). For this study, the phrasing of the items was changed from the past to the future tense to measure the intention of the respondent to conduct each of the six gatekeeper behaviors, rather than what has already been done. High mean scores indicated a strong intention to perform a gatekeeper role. The observed Cronbach’s α was 0.78, which is considered within the acceptable range (47). The results of the CFA supported the construct validity of the intention measure, demonstrating acceptable model fit (CFI = 0.952, TLI = 0.920, RMSEA = 0.096, SRMR = 0.037).

### Data analysis plan

Preliminary and bivariate analyses were conducted using SPSS version 25.0 (61). Mplus version 8.3 (50) was used for the path analysis. Before the analyses, the newly translated scale was assessed for reliability and construct validity.

#### Bivariate analyses

A *t*-test and one-way ANOVA were conducted to determine the group differences in the level of gatekeeper knowledge, attitudes, perceived preparedness, and intention according to demographic variables. Correlations were examined between all model variables, and the assumption of non-multicollinearity was met, as evidenced by the largest absolute value for the bivariate correlation being 0.38.

#### Path analysis

To test the hypothesized path model presented in Figure 1, a path analysis was conducted to examine the relationship between suicide educational experiences, suicide competencies (including knowledge, attitudes, and perceived preparedness), and the intention to perform a gatekeeper role. In this regard, (1) to assess the fit of the hypothesized path model in the current sample, several fit indices were used.^4^ (2) The significance and magnitude of the path coefficients between educational experiences and gatekeeper competencies were then examined to address Hypothesis 2. In addition, the total and indirect effects of suicide-related educational experiences on suicide competencies and intention to perform a gatekeeper role were tested. (3) To address Hypothesis 3, the path coefficient between educational experience/gatekeeper competencies and gatekeeper intentions was examined using the same process as in Step 2.

## Results

### Descriptive analyses

The results from descriptive analyses of educational experiences about suicide are presented in Table 2. In class, 65.7% (*n* = 326) of participants had learned about suicide at least once. The most commonly covered topic was warning signs of suicide (46.6%, *n* = 231), followed by theories on suicide (35.1%, *n* = 174). With field experiences, 24.2% (*n* = 120) of participants had learned about suicide. Suicide warning signs (18.3%, *n* = 91) were the most often discussed, followed by suicide risk assessment (12.1%, *n* = 60). Only 7.7% (*n* = 38) of participants reported having contact with suicidal clients in the field.

**Table 2:** Descriptive statistics of educational experience about suicide.

Responses		*N*	*%*
Learned about suicide in class	Yes	326	65.7
	No	170	34.3
The contents	Suicide risk assessment	158	31.9
	Warning sings	231	46.6
	Referral	94	19.0
	Short-term intervention	128	25.8
	Suicide prevention policies	57	11.5
	Theories on suicide	174	35.1
Learned about suicide at field	Yes	120	24.2
	No	376	75.8
The contents	Suicide risk assessment	60	12.1
	Warning sings	91	18.3
	Referral	53	10.7
	Short-term intervention	37	7.5
	Suicide prevention policies	34	6.9
	Theories on suicide	65	13.1
Experienced suicidal clients at field	Yes	38	7.7
	No	458	92.3

### Bivariate analyses

Independent samples t-tests were conducted to see whether there were significant mean differences in knowledge, attitudes, perceived preparedness, and intention scores according to gender and school year, as presented in Table 1. The results showed that females (*M* = 63.42) had significantly higher knowledge scores than males (*M* = 59.90, *t* = −2.299, *p* < 0.05). The results also showed that males (*M* = 4.57) scored significantly higher in Perceived Preparedness than females (*M* = 4.17, *t* = 3.719, *p* < 0.001). The mean scores for the third and fourth-year students were not significantly different for any of the measures.

One-way ANOVA was used to test the group differences in Knowledge, Attitudes, Perceived Preparedness, and Intention according to family income. As presented in Table 1, a significant mean difference in attitudes was found (*F* = 5.660, *p* < 0.001) between groups with different subject levels of family income. Post-hoc Tukey tests showed that those who reported they are in the upper class (*M* = 23.25) scored significantly lower than those in the upper middle (*M* = 32.71, *p* < 0.05), middle class (*M* = 34.69, *p* < 0.01), lower middle class (*M* = 34.99, *p* < 0.01) and low class (*M* = 35.29, *p* < 0.01), indicating more positive attitudes toward suicide prevention among individuals in the upper class than any other groups. A significant effect was found from the main analysis for intention (*F* = 3.197, *p* < 0.05), but Post-hoc Tukey tests indicated no significant group differences.

A one-way ANOVA was also conducted to examine the group effect of subjective mental health status. Significant group differences were found only for attitudes (*F* = 8.73, *p* < 0.001). Post-hoc Tukey tests revealed that those who rated their mental health status as ‘*Very Good*’ (*M* = 31.28) scored significantly lower on ATTSP compared to those with ‘Average’ (*M* = 35.17, *p* < 0.001), ‘Poor’ (*M* = 37.07, *p* < 0.001), and ‘Very Poor’ mental health (*M* = 37.83, *p* < 0.01), indicating more positive attitudes toward suicide prevention among individuals with “*Very good*” mental health than the other three groups.

Table 3 presents the results from correlation analyses along with descriptive statistics for model variables. The correlations between all model variables were statistically significant (*p* < 0.05) except for between the field experiences and the level of gatekeeper knowledge and between the level of gatekeeper knowledge and preparedness. All significant correlations were in a positive direction except for the correlations involving attitudes toward suicide prevention, which indicate that increased suicide education experiences in class and the field, as well as suicide prevention competencies, were related to the decreased level of negative attitudes toward suicide prevention.

**Table 3:** Descriptive statistics and correlation.

Variables	Class	Field	Knowledge	Attitude	Preparedness	Intention
Class	1.00					
Field	0.36^**^	1.00				
Knowledge	0.11^*^	0.06	1.00			
Attitude	−0.09^*^	−0.15^**^	−0.27^**^	1.00		
Preparedness	0.20^**^	0.27^**^	−0.001	−0.24^**^	1.00	
Intention	0.13^**^	0.13^**^	0.27^**^	−0.26^**^	0.38^**^	1.00
Range	0–6	0–6	0–100	14–70	1–7	1–5
Mean(SD)	1.70(1.70)	0.68(1.42)	62.64(14.19)	34.40(6.22)	4.26(1.01)	3.83(0.55)

Class = Having received suicide education at class. Field = Having received suicide education outside of class, including field practicum. ^*^p < 0.05; ^**^p < 0.01.

### Path analyses

#### Assessing the model fit of the hypothesized path model

The path model was found to fit well with the data, as indicated by examining the fit indices. Although the result from the chi-square test was significant (χ^2^ = 4.108, *df* = 3, *p* < 0.05), the CFI and TLI values were above the threshold for an acceptable fit (CFI = 0.995, TLI = 0.977), and the RMSEA and SRMR values were below the threshold for a reasonable error approximation (RMSEA = 0.027, SRMR = 0.022).

#### Effects of educational experiences on suicide prevention competencies

The results of the path analyses are presented in Table 3 and Figure 1. Both in-class and field-related learning experiences significantly affected suicide prevention competencies but through different paths. Specifically, education about suicide in the classroom significantly improved gatekeeper knowledge (*β* = 0.103, *p* < 0.05) and perceived preparedness (*β* = 0.118, *p* < 0.01) but did not have a significant effect on attitudes toward suicide prevention. In contrast, field experiences related to suicide predicted a less negative attitude toward suicide prevention (*β* = −0.128, *p* < 0.01) and increased perceived preparedness (*β* = 0.194, *p* < 0.001) but did not have a significant effect on knowledge about suicide.

**Figure 2:**
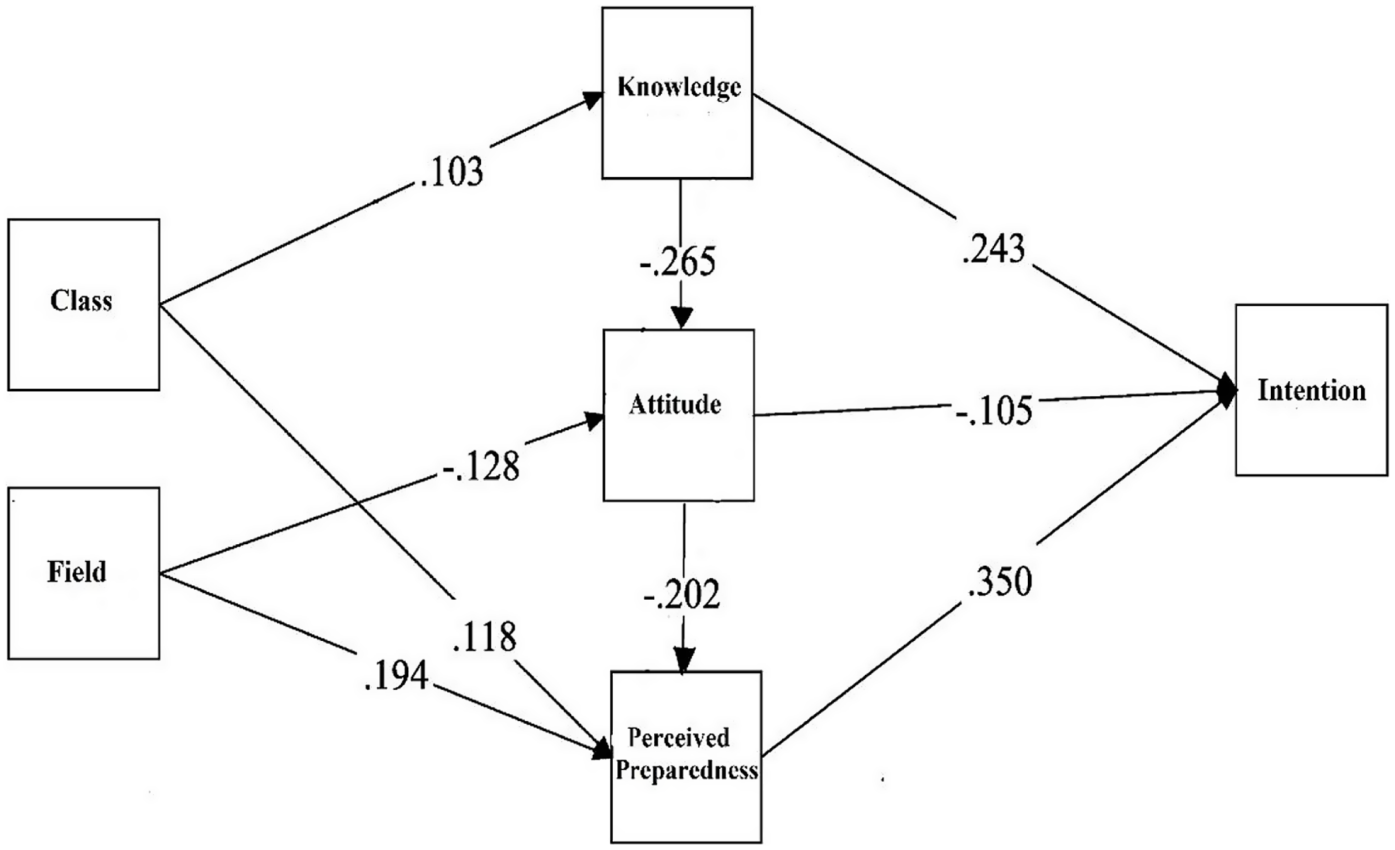
The path model of intent to perform gatekeeper roles (with estimates of significant standardized path coefficients).

The results of the study also revealed that suicide prevention competencies were indirectly related to suicide education through the mediation of other components of the competencies. As presented in Table 3, the total effect of field education on perceived preparedness was significant (ꞵ = 0.221, *p* < 0.001), which included the indirect effects (ꞵ = 0.027, *p* < 0.05) via attitude. Specifically, individuals who received additional education about suicide in the field were more likely to perceive themselves as better prepared to perform gatekeeper roles through improved attitudes. However, the indirect effects of class education on preparedness through knowledge and attitude were not found to be significant. Although the total effects of the class on attitude were not significant, the indirect effect through knowledge was significant (ꞵ = −0.027, *p* < 0.05). Learning about suicide in class only indirectly decreased negative attitudes toward suicide education through the increased level of knowledge about suicide (see Table 4 and Figure 2).

**Table 4:** Direct, indirect, and total effects on knowledge, aptitudes, perceived preparedness, and intention for gatekeeper role.

Outcome variable	Predictor variable	Total effects	Direct effects	Indirect effects
Intention	Class	0.073^**^	N/A	0.073^**^
	Knowledge			0.025^*^
	Attitude			0.001
	Preparedness			0.041^*^
	Knowledge → Attitude			0.003
	Attitude → Preparedness			0.001
	Knowledge → Attitude → Preparedness			0.002
	Field	0.097^***^	N/A	0.097^***^
	Knowledge			0.005
	Attitude			0.013
	Preparedness			0.068^***^
	Knowledge → Attitude			0.001
	Attitude → Preparedness			0.009^*^
	Knowledge → Attitude → Preparedness			0.000
	Knowledge	0.290^***^	0.243^***^	0.047^**^
	Attitude			0.028^*^
	Attitude → Preparedness			0.019^***^
	Attitude	−0.176^***^	−0.105^*^	−0.071^***^
	Prepared			−0.071^***^
	Preparedness	0.350^***^	0.350^***^	N/A
Perceived	Class	0.126^**^	0.118^**^	0.008
Preparedness	Attitude			0.003
	Knowledge → Attitude			0.006
	Field	0.221^***^	0.194^***^	0.027^*^
	Attitude			0.026^*^
	Knowledge → Attitude			0.001
	Knowledge	0.054^***^	N/A	0.054^***^
	Attitude			0.054^***^
	Attitude	−0.202^***^	−0.202^***^	N/A
Attitude	Class	−0.040	−0.013	−0.027^*^
	Knowledge			−0.027^*^
	Field	−0.134^**^	−0.128^**^	−0.006
	Knowledge			−0.006
	Knowledge	−0.265^***^	−0.265^***^	N/A
Knowledge	Class	0.103^*^	0.103^*^	N/A
	Field	0.022	0.022	N/A

^*^p < 0.05; ^**^p < 0.01; ^***^p < 0.001.

#### Effects of educational experiences and suicide competencies on intent to perform gatekeeper

Among components of suicide prevention competencies, perceived preparedness had the strongest relationship with participants’ intention to perform gatekeeper roles (ꞵ = 0.350, *p* < 0.001). Additionally, a higher level of knowledge (ꞵ = 0.243, *p* < 0.001) and diminished negative attitudes towards suicide prevention (ꞵ = −0.105, *p* < 0.05) were also significantly associated with the intention to perform gatekeeper roles. The results also revealed that the intention to perform gatekeeper roles was indirectly predicted by knowledge through attitudes (ꞵ = 0.028, *p* < 0.05) and perceived preparedness via attitude (ꞵ = 0.019, *p* < 0.001). Attitudes also indirectly predicted the intention through perceived preparedness (ꞵ = −0.071, *p* < 0.001).

Suicide education indirectly increased the intention to perform gatekeeper roles through the mediation of improved suicide competencies. Specifically, the educational experiences in the classroom had significant indirect effects on intention (ꞵ = 0.073, *p* < 0.01) through knowledge (ꞵ = 0.025, *p* < 0.05) and preparedness (ꞵ = 0.041, *p* < 0.05). Similarly, the level of field education also had significant indirect effects on intention (ꞵ = 0.097, *p* < 0.001), through the perceived preparedness (ꞵ = 0.068, p < 0.001), as well as perceived preparedness via attitude (ꞵ = 0.009, *p* < 0.05).

## Discussion

The proposed model of the pathway to intention to perform gatekeeper roles, formulated based on the current gatekeeper training literature, fits well among social work students in Korea. Significant relationships were found between the educational experiences about suicide and the intention to perform gatekeeper roles. As hypothesized, these relationships were mediated by suicide prevention competencies, including knowledge, attitude, and perceived preparedness.

Suicide-related educational experiences improved gatekeeper competencies among social work students, but different pathways were found according to the learning venue. Learning in class was associated with an improved level of knowledge but not with positive attitudes toward suicide prevention. On the contrary, the field experiences were associated with positive attitudes toward suicide prevention but not with improved knowledge. The results suggest that in order to enhance suicide competencies comprehensively, diverse educational opportunities should be provided. The education provided in the field likely contains more interactive and skill-based learning opportunities, such as direct contact with the clients or learning activities for skill acquisition, than in-class learning. The findings support the prior gatekeeper training literature emphasizing the importance of adapting diverse learning strategies instead of relying on didactic teaching strategies in order to ensure the effectiveness of the training (4, 51, 52). The challenges that are continuously reported in efforts to improve and maintain positive attitudes toward suicide prevention (5, 23) underscore the promise of the current findings on the effectiveness of field education in improving such attitudes, particularly for social work education.

Social work students’ suicide competencies, knowledge, attitudes, and perceived preparedness significantly predicted their intention to practice as gatekeepers. These results support the central concept of the theory of intention (58) and the gatekeeper training literature, which emphasizes the significance of key competencies in effective suicide prevention (23, 32). Moreover, given the challenges in measuring practice behavior, especially due to limited opportunities for direct contact with clients among students, the significant correlations between suicide prevention competencies and intention suggest the potential usefulness of these constructs in measuring educational outcomes and readiness as gatekeepers.

The perception of preparedness emerged as the most strongly associated core competency with intention and also acted as a mediator of the relationship between other competency measures and intention. These findings align with the principles of social cognitive theory (53), which propose that an individual’s self-efficacy in performing a behavior in a specific situation directly predicts their practice behaviors and serves as a mediator of applying knowledge to behavior. In this study, improvements in knowledge and attitudes toward suicide prevention increased students’ intention to perform gatekeeper roles by enhancing their perception of preparedness. Given that perceived readiness can be subjective and personal, educators should consider strategies to boost students’ confidence in learning outcomes. As predicted, the study results showed that suicide competencies mediated the relationship between educational experiences during college and the intention to perform gatekeeper roles among social work students. Including suicide-related content in the curriculum increased the intention to perform gatekeeper roles through increased knowledge and perceived preparedness. On the other hand, educational experiences in the field impacted intention through improved attitudes and perception of preparedness. While most recent gatekeeper literature adopts a competencies-based approach (23, 32), the lack of empirical evidence for how these competencies interact with each other to influence practice behavior has been an ongoing concern (23, 30). By demonstrating the pathways from education to behavioral intention through the core competencies, the current study can make an important contribution to filling this gap.

### Limitations

Several limitations should be considered when interpreting the results of this study. First, using cross-sectional data limits the ability to establish causal relationships between educational experiences/core competencies and the behavioral intention to act as a gatekeeper. Instead, the observed relationships should be interpreted as associations. Second, although the sample of social work students in this study was nationally representative, the response rate from the online survey was 34%. Typically, the average response rate for an online survey is lower than for offline surveys (54). The low response rate in the online survey may have led to an overestimation of students’ knowledge and perceptions about suicide prevention, as those with a particular interest in the topic may have been more likely to participate. Third, the survey was conducted during the fall semester of 2020, when the COVID-19 pandemic was ongoing. This could have reduced students’ opportunities for hands-on experiences related to suicide risk due to limited direct contact with clients.

This study has several limitations regarding the measures. First, educational experiences were assessed using only two items because of the lack of established measures for educational experiences regarding the gatekeeper role. We created the checklist based on key research; however, it is less comprehensive and robust than previously validated instruments. Second, although the gatekeeper competency measures were translated into the Korean version following an established process, we only presented the preliminary evidence for construct validity with the results of the CFA. Without established Korean measures for direct comparison, we could not further confirm criterion validity. Consequently, measurement errors cannot be entirely ruled out when interpreting the current results. Third, the knowledge, perceived preparedness, and intention measures regarding gatekeeper roles were initially developed for secondary school staff (42). Although these measures demonstrated the applicability in assessing gatekeeper competencies among social work students in the US (20), differences in school and social work settings should be noted. Future research should consider validating the measures before applying them to diverse populations of gatekeepers. Finally, the measurement of educational experiences was limited to a quantitative survey that focused on whether students had ever learned about specific suicide-related content. Consequently, this study did not capture the utilization of teaching strategies such as role-playing or the quality of interactions between lecturers and students. Future studies should consider qualitative differences in students’ educational experiences by assessing how effectively the content was delivered through more experiential teaching methods.

### Implications for future studies and social work education

The results underscore the need to integrate suicide-related content into the social work curriculum to prepare prospective social workers to serve as professional gatekeepers. Students with more educational experiences related to suicide have demonstrated higher readiness to act as gatekeepers. These findings suggest that social work education has the potential to prepare students as professional gatekeepers by improving their knowledge, attitudes, confidence, and behavioral intentions. Specifically, the concept of a “professional gatekeeper” can be helpful when discussing the roles of social workers in suicide prevention who do not work in the mental health field but in various community settings.

When incorporating suicide prevention efforts into university courses or training, increasing competencies should be a key objective of education. Suicide competencies mediated the positive impacts of suicide education on the behavioral intention of gatekeepers, indicating that participation in training or classes alone is insufficient for training effective gatekeepers. Therefore, diverse educational strategies should be adopted to enhance competencies comprehensively. For example, interactive learning, such as role-playing, small and large group discussions, training cases, and expert demonstrations, has been recommended as an effective alternative to didactic-only teaching strategies in studies assessing the effectiveness of gatekeeper training (4, 55). Moreover, skills-based training in assessment and risk management, active listening, and communication skills are emphasized as key components to enhance competencies (21, 52). These core skills of gatekeepers are highly relevant to general social work practice and can be effectively integrated into traditional social work practice courses.

To promote positive attitudinal changes toward suicide prevention, social work educators should prioritize providing field experiences. In the current study, diverse educational experiences through fieldwork or community activities, rather than in-class learning, predicted positive attitudes toward suicide prevention. Attitudinal changes are often considered the core component of competencies, but they can also act as barriers to transferring educational outcomes to practical behaviors. For instance, interpersonal discomfort or ambivalence about addressing suicide, beliefs in suicide myths, inaccurate beliefs about treatment, and concerns about betraying trust can all hinder the performance of gatekeeper roles (5). In addition to encouraging students to participate in diverse field experiences, modifications to the suicide-related course content, such as including content about stigma toward suicide and mental health treatment, should also be considered.

Considering that a significant proportion of students had no prior opportunities to learn about suicide, curriculum changes should be considered to ensure all social work graduates are exposed to essential educational content related to suicide. For example, the BSW courses can focus on covering knowledge of suicide risk factors, warning signs, and the referral process and incorporate diverse learning opportunities that can help students form positive attitudes and beliefs toward suicide prevention. Furthermore, the content addressed in MSW can be expanded to include clinical practice skills to work with those at risk and strengthen basic competencies with a more specialized understanding of suicide among the population as related to students’ concentration. These changes would present an initial step for social work education to undertake their responsibility in more actively addressing suicide prevention.

Future studies should utilize a longitudinal study design to establish causal relationships between education, competencies, behavioral intention, and actual practice behavior. While the current study was based on the assumption from the theory of intention, empirical evidence linking behavioral intention and gatekeeper behavior is lacking. Empirical support for behavioral intention as a proximal outcome of practice behavior will help efficacy studies of gatekeeper education or suicide-related courses among students or future practitioners. Incorporating longitudinal studies that analyze actual practice behavior would ultimately enable us to establish a direct correlation between educational efforts in suicide prevention and reducing suicide rates.

Although gender differences were not the focus of our study, our preliminary analyses revealed gender differences in suicide competencies, which warrant further investigation. The female students’ knowledge level was higher, while the male students demonstrated higher perceived readiness as gatekeepers. Future studies should investigate the mechanisms underlying these gender differences, as they reflect distinct educational needs across genders.

Studying the adaptability of existing gatekeeper training, such as QPR (Question, Persuade, Refer) or ASIST (Applied Suicide Intervention Skills Training), for social work students would be helpful. Despite the lack of educational opportunities among social work students, the current opportunities available through universities have helped enhance suicide prevention competencies among future social workers. As we work towards preparing social work students as professional gatekeepers by upgrading the current social work course, we should also consider adapting established training programs in social work education. However, since the contents and lengths of the training programs are diverse, future studies are needed to test which training program is most suitable and adequate for social work students.

## Conclusion

In conclusion, social work students with more educational experiences about suicide had higher competencies and intentions to perform gatekeeper roles. These findings highlight the importance of preparing social work students as professional gatekeepers by providing various educational experiences and thus improving the critical competencies of gatekeepers. By doing so, the academia of social work education can contribute to addressing the pressing public health issue of suicide. Further research is needed to examine the long-term impact of such educational efforts on gatekeeper behaviors and suicide prevention outcomes.

## Data Availability

The raw data supporting the conclusions of this article will be made available by the authors, without undue reservation.

## Footnotes

^1^According to the Korean Association of Social Workers (18), more than 82.5% of social workers in Korea have a BSW as a final degree.

^2^The average response rates of online surveys are lower than those of offline surveys. Meta-analysis revealed % an average response rate of 34% (54). A recent online survey for Korean university students reported a response rate of 22% (56).

^3^During the first 2 years of their program, students are required to take introductory courses that serve as prerequisites for the field practicum.

^4^A non-significant chi-square test suggests a good fit. An acceptable fit is indicated by CFI and TLI values of 0.90 or above, and values above 0.95 suggest a good fit (48, 59, 60). A reasonable error approximation is suggested by an SRMR of 0.08 or less and a RMSEA of 0.06 or less (48).
